# Clinical characteristics and long-term evolution of lamellar macular hole in high myopia

**DOI:** 10.1371/journal.pone.0232852

**Published:** 2020-05-06

**Authors:** Yun Hsia, Tzyy-Chang Ho, Chang-Hao Yang, Yi-Ting Hsieh, Tso-Ting Lai, Chung-May Yang

**Affiliations:** 1 Department of Ophthalmology, National Taiwan University Hospital, Taipei, Taiwan; 2 College of Medicine, National Taiwan University, Taipei, Taiwan; 3 Graduate Institute of Clinical Medicine, College of Medicine, National Taiwan University, Taipei, Taiwan; National Yang-Ming University Hospital, TAIWAN

## Abstract

**Purpose:**

To evaluate the clinical characteristics and evolution of lamellar macular hole (LMH) in high myopia and the parameters associated with structural worsening, defined as the development of foveal detachment or full-thickness macular hole.

**Methods:**

Patients with high myopia and LMH were retrospectively recruited. The clinical characteristics and various parameters of optical coherence tomography were identified at baseline and during follow-up visits. Cox regression analysis was used to evaluate the hazard ratios for foveal detachment and full-thickness macular hole.

**Results:**

Among 112 eyes (98 patients), 64.3% were female; the mean axial length of all eyes was 29.6 ± 1.9 mm. The ‘LMH without retinoschisis’ group accounted for 39.3% of the eyes. Forty-two percent developed structural worsening within a median follow-up of 67 months. Multivariable regression on all cases showed elevated tissue inside the LMH (*P* = 0.003) protected against structural worsening while V-shaped LMH (*P* = 0.006) predicted it. In the “LMH with retinoschisis group”, ellipsoid zone disruption (*P* = 0.035), and V-shaped LMH (*P* = 0.014) predicted structural worsening, while elevated tissue inside the LMH (*P* = 0.028) protected against it. In the “LMH without retinoschisis group”, no associated factor was identified.

**Conclusions:**

LMHs in high myopia are unstable, especially those with V-shaped LMH. Elevated tissue inside LMHs have a protective effect against further structural worsening.

## Introduction

Lamellar macular hole (LMH) is a common macular structural abnormality in patients with high myopia (4.8%–20.7%) [1, 2]. In highly myopic eyes, the clinical course of LMH may be more complicated than that in idiopathic LMH because the adherent vitreous cortex and epiretinal membrane exert a complex anteroposterior and tangential traction, and because the internal limiting membrane (ILM) is rigid and posterior staphyloma is often present [3–5]. Some LMHs may stay unchanged or show minimal progression for many years, while others may progress to full-thickness macular hole (FTMH) or macular hole with retinal detachment (MHRD), causing severe vision loss [6–8]. Studies on myopic traction maculopathy (MTM) in general have shown that, when foveal retinoschisis advances to retinoschisis with foveal detachment (FD), the probability of FTMH formation is very high [2]. However, few investigations have addressed the evolution of LMH in high myopia specifically. Furthermore, in highly myopic eyes, LMH may exist alone or in association with retinoschisis [3, 5, 9], and it is unclear whether these two different types of LMH exhibit different clinical characteristics and outcomes.

In the present study, we retrospectively reviewed LMH in highly myopic eyes and examined lesion evolution. By analyzing the various parameters related to progression, we aimed to identify the risk and protective factors associated with structural worsening. This may inform decisions regarding follow-up strategy and timing of surgery.

## Materials and methods

### Study population

From January 2010 to December 2017, consecutive cases of high myopia with LMH were retrospectively reviewed. Regardless of whether it was associated with retinoschisis, LMH was diagnosed using the following updated criteria proposed by the International Vitreomacular Traction Study Group: (1) irregular foveal contour; (2) defect in the inner fovea (with or without actual loss of tissue); (3) separation between the inner and outer retinal layers in the fovea; (4) absence of full-thickness foveal defect [10]. High myopia was defined as a spherical equivalent refractive error more severe than -6.0 diopters and/or an axial length ≥ 26 mm. LMHs were identified using spectral-domain optical coherence tomography (SD-OCT) (RTVue Model-RT 100 scanner, version 3.5: Optovue Inc., Fremont, CA, USA). Only patients with a follow-up of at least 2 years were included. Those with a history of RD, vascular or inflammatory disease, or previous intraocular surgery other than cataract operation were excluded. The study was approved by the Institutional Review Board at National Taiwan University Hospital and followed the tenets of the Declaration of Helsinki. The informed consent was waived.

### Clinical characteristics and OCT parameters

Demographics and ocular biometry were recorded. Best-corrected visual acuity (BCVA) was measured using a Snellen chart and converted to logarithm of the minimal angle of resolution values for statistical analysis. The post-operative visual acuity was measured at least 6 months after the surgery. Structural worsening was defined as the development of FD or FTMH. The time to structural worsening was documented, as were the surgical methods and results. Macular structures were regularly examined using slit-lamp biomicroscopy, fundus photography, and OCT every 3 to 6 months.

Based on the new grading system [11], the severity of myopic atrophic maculopathy was classified as follows: A0, no myopic retinal lesions; A1, tessellated fundus; A2, diffuse chorioretinal atrophy; A3, patchy chorioretinal atrophy; and A4, macular atrophy. Eyes with A2 or higher-grade retinal lesions were considered to have myopic atrophic maculopathy.

**Fig 1:**
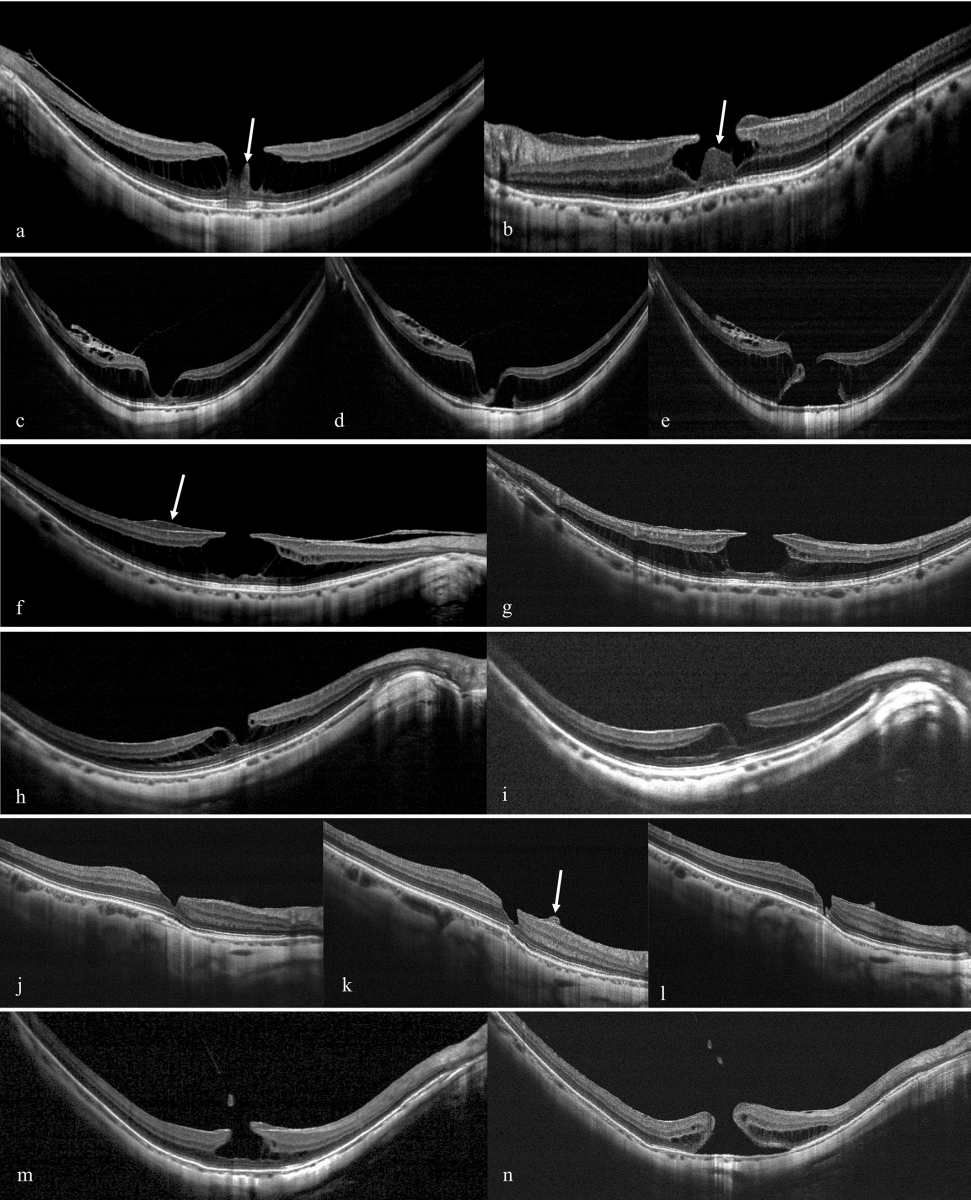
(a-b) Two eyes with elevated tissue inside a lamellar macular hole (LMH) (arrow). The evolution of LMHs in the ‘LMH with retinoschisis group’: (c-e) The first case had a V-shaped LMH with extensive retinoschisis. The LMH progressed into foveal detachment after 6 months, and further progressed into full-thickness macular hole (FTMH) about 4 months later. (f-g) The second case had an A-shaped LMH with retinoschisis. The LMH remained stable during the 44-month follow-up. Lamellar-associated epiretinal proliferation was noted around the opening of LMH (arrow). (h-i) The third case had parafoveal retinoschisis with a V-shaped LMH. The LMH remained stable during the 44-month follow-up. The evolution of LMHs in the ‘LMH without retinoschisis group’: (j-l) The fourth case had a V-shaped LMH. Lamellar-associated epiretinal proliferation developed around the LMH (arrow). During the 15-month follow-up, the LMH deepened progressively and developed into a FTMH with a wide opening. (m-n) The fifth case had an A-shaped LMH. Optical coherence tomography showed evidence of previous vitreomacular traction with a piece of avulsed retinal tissue. It progressed to FTMH 21 months later. This narrow-opening pattern was similar to an idiopathic FTMH.

Standard 8-mm horizontal and vertical OCT scans, centered on the fovea, were taken using the registration function. The following OCT parameters were documented or measured using the manual caliper function of the built-in software: central retinal thickness (CRT), subfoveal choroidal thickness, maximal horizontal and vertical LMH diameters, and minimal residual foveal thickness. The minimal residual foveal thickness was measured as the shortest distance from the base of the LMH to the Bruch’s membrane at the fovea. The OCT images were also examined for the presence of lamellar hole-associated epiretinal proliferation [12], macular retinoschisis, epiretinal membrane, staphyloma, vitreomacular traction, and elevated tissue inside the LMH (Fig 1A and 1B); the integrity of the ellipsoid zone was also assessed.

The presence of retinoschisis at the macula was recorded at baseline and at follow-up visits. The type of LMH was determined at baseline. The ‘LMH with retinoschisis’ group had both LMH and macular retinoschisis (Fig 1C–1I), while the ‘LMH without retinoschisis’ group had no macular retinoschisis at baseline (Fig 1J–1N). The extent of retinoschisis was categorized as follows, according to Shimada’s classification [13], as either parafoveal retinoschisis, which involved foveal retinoschisis that did not cover the entire macula (Fig 1h), or extensive retinoschisis, which involved the entire macula (Fig 1C and 1F). Macular retinoschisis was further classified into inner or outer types. Eyes with outer macular retinoschisis had intraretinal splitting in or outer to the outer plexiform layer. Eyes with inner macular retinoschisis had intraretinal splitting inner to the outer plexiform layer [8].

LMHs were classified as either V-shaped (Fig 1C, 1H and 1J) or A-shaped (Fig 1F and 1M). V-shaped LMHs had a smaller diameter intraretinally than at the retinal surface, while A-shaped LMHs had a larger diameter intraretinally.

### Surgical criteria and technique

During follow-up, operation would be suggested to patients who suffered worse vision associated with progression of the foveal structural changes, FD, FTMH, or RD. If the patient agreed to receive operation after pros and cons of the procedures were explained, vitrectomy would be arranged within one month based on the severity of the condition. In brief, a standard three-port 23-gauge or 25-gauge pars plana vitrectomy was performed by three retinal specialists (C.M.Y. & T.C.H. & C.H.Y). Triamcinolone acetonide-assisted posterior hyaloid separation and removal were performed, followed by epiretinal membrane removal with microforceps if identified. Indocyanine green dye-assisted ILM peeling was then performed within the arcade. For cases without FTMH, fovea-sparing ILM peeling technique was adopted [14]. For cases with FTMH, temporal inverted ILM flap technique [15] or inverted ILM flap insertion technique [16] was performed depending on the condition. The flap was stabilized by copious amount of Viscoat (Alcon Laboratories, Fort Worth, TX). Finally, air-fluid exchange was done. The vitreous cavity was flushed with 15% perfluoropropane (C3F8) in those cases with FTMH. Rarely, silicone oil tamponade was used at the surgeons’ discretion.

### Statistical analysis

All statistical analyses were performed using RStudio (version 3.6.0; RStudio, Inc, Boston, MA, USA). For descriptive statistics, mean and standard deviation were calculated for parametric data, while median and range were calculated for non-parametric data. Percentages were calculated for categorical variables. Changes in clinical parameters between enrollment and final follow-up were evaluated using paired t-tests. To identify predictive factors for the development of FTMH or FD, Cox proportional hazards regression analysis was performed. Schoenfeld residuals were obtained to test the proportional hazard assumption of the Cox regression. Variable with *P*-values *<* 0.1 in univariable Cox proportional hazards regression analysis was included in multivariable Cox proportional hazards regression analysis. For the subgroup analysis of eyes with different extent of retinoschisis and LMH shapes, Chi-squared test or Fisher’s exact test was used for categorical variables and independent t-test was used for numerical variables. *P*-values < 0.05 were considered statistically significant.

## Results

One hundred and twelve eyes of 98 patients (26 men and 72 women) were enrolled. The patients’ mean age was 61.8 ± 9.4 years. Their mean axial length was 29.6 ± 1.9 mm and their BCVA at baseline was 0.42 ± 0.35. At presentation, 44 eyes (39.3%) were in the ‘LMH without retinoschisis’ group, while 68 (60.7%) were in the ‘LMH with retinoschisis’ group. There were more women in the ‘LMH with retinoschisis’ group. Eyes in the ‘LMH with retinoschisis’ group had worse baseline BCVA, more staphyloma, longer axial length, thicker CRT, thinner subfoveal choroidal thickness, and deeper LMH vertical extension (Table 1). The shape of LMH showed no association with the presence of retinoschisis (*P =* 0.223 by Chi-squared test), severity of myopic atrophic maculopathy (*P =* 0.999 by Chi-squared test), or visual acuity (*P =* 0.1 by t-test).

**Table 1:** The baseline clinical characteristics.

Baseline characteristics	All (112)	LMH without retinoschisis (44)	LMH with retinoschisis (68)	*P* value
**Age (years)**	61.8 ± 9.4	63.5 ± 9.5	60.7 ± 9.2	0.118 ^a^
**Sex (female)**	72 (64.3)	26 (59.1)	56 (82.4)	**0.013** ^b^
**Axial length (mm)**	29.6 ± 1.9	29.1 ± 2.0	30.0 ±1.7	**0.020** ^a^
**Visual acuity ^c^**	0.42 ± 0.35	0.33 ± 0.32	0.48 ± 0.37	**0.023** ^a^
**Epiretinal membrane**	67 (59.8)	24 (54.5)	43 (63.2)	0.472 ^b^
**LHEP**	48 (42.9)	22 (50.0)	26 (38.2)	0.302 ^b^
**Staphyloma**	92 (82.1)	28 (63.6)	64 (94.1)	**<0.001** ^b^
**Ellipsoid zone disruption**	44 (39.3)	18 (40.9)	26 (38.2)	0.923 ^b^
**Vitreomacular traction**	20 (17.8)	10 (22.7)	10 (14.7)	0.407 ^b^
**Elevated tissue inside the LMH**	36 (32.1)	15 (34.1)	21 (30.9)	0.882 ^b^
**Vertical diameter of LMH (μm)**	250.9 ± 110.3	174.2 ± 77.5	300.5 ± 99.6	**<0.001**^a^
**Horizontal diameter of LMH (μm)**	320.7 ± 158.1	329.0 ± 147.2	315.4 ± 165.7	0.651 ^a^
**Central retinal thickness (μm)**	384.1 ± 119.9	303.5 ± 79.4	436.2 ± 112.9	**<0.001** ^a^
**Residual foveal thickness (μm)**	130.6 ± 43.6	127.7 ± 34.3	132.5 ± 48.8	0.544 ^a^
**Subfoveal choroidal thickness (μm)**	57.2 ± 36.0	71.7 ± 45.0	46.2 ± 26.0	**0.032** ^a^

Mean ± SD; N (%); LHEP, lamellar hole-associated epiretinal proliferation; LMH, lamellar macular hole

^a^ t-test

^b^ Chi-squared test

^c^ LogMAR VA, logarithm of the minimum angle of resolution visual acuity

**Fig 2:**
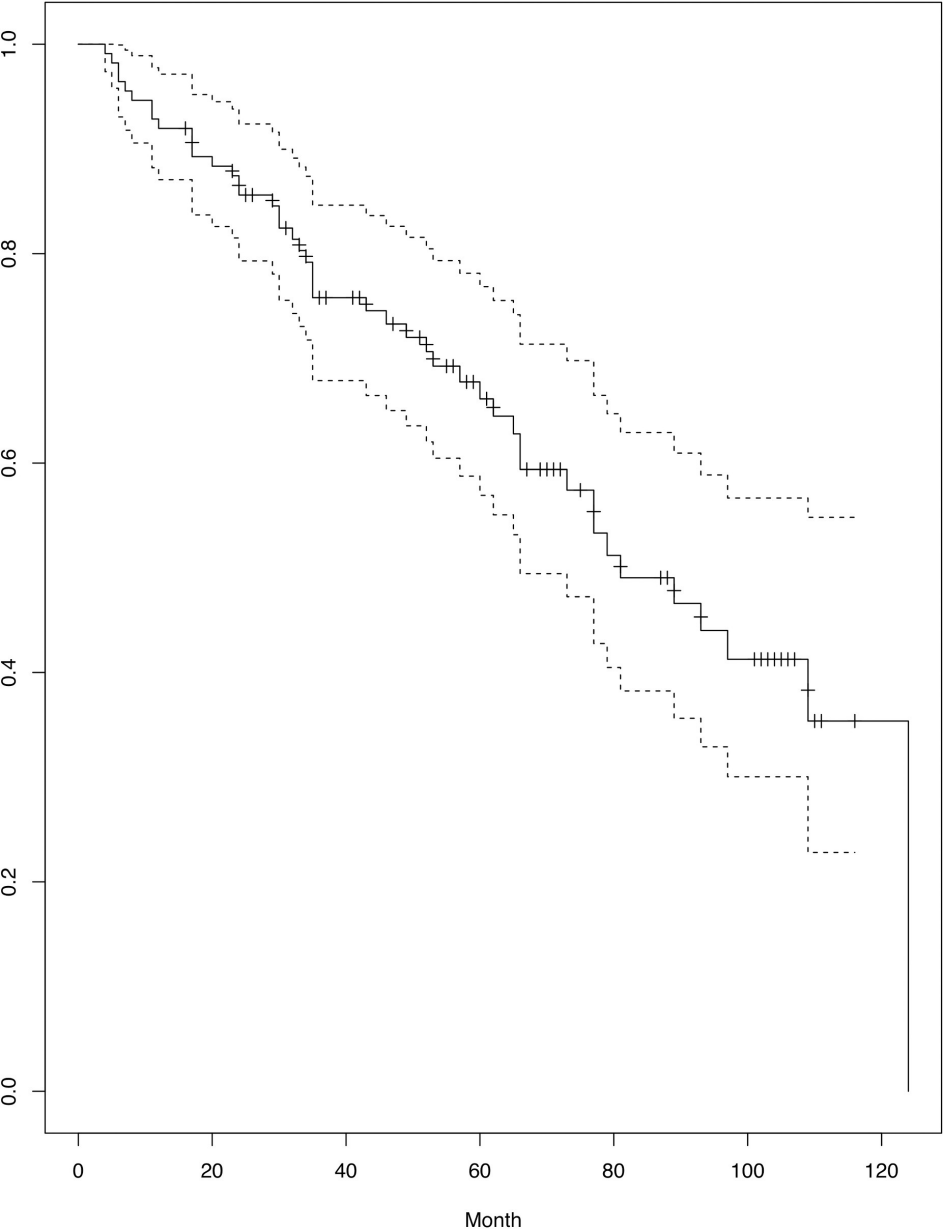
The FTMH- and FD-free survival curve. The hazard of structural worsening was similar at each time point.

During a median follow-up of 67 months (range: 24–146 months), the LMH diameters increased both vertically (250.9 ± 110.3 μm vs. 296.7 ± 142.8 μm, *P* < 0.001 by paired t-test) and horizontally (320.7 ± 158.1 μm vs. 357.4 ± 170.6 μm, *P* = 0.011). The CRT increased (384.1 ± 119.9 μm vs. 420.8 ± 141.0 μm, *P* < 0.001), while the residual foveal thickness decreased (130.6 ± 43.6 μm vs. 120.9 ± 44.1 μm, *P* = 0.031). Nine eyes (20.5%) in the ‘LMH without retinoschisis’ group developed new macular retinoschisis. Fig 2 shows the FTMH- and FD-free survival curve. The hazard was similar at each time point, indicating that both FD and FTMH could occur at any time point. Sixty-five eyes (58.0%) had neither FD nor FTMH, while 15 eyes (13.4%) developed FD. Four eyes (3.6%) had FD first and developed FTMH subsequently, and two had MHRD. Twenty-eight eyes (25.0%) developed FTMH and nine had concurrent FTMH and MHRD (Table 2). Forty-seven eyes met our surgical criteria. Two of them had spontaneous resolution before operation. Operations were then performed on 45 eyes, of which 41 eyes (91%) showed anatomical success after surgery and 4 eyes (9%) showed persistent FTMH. Those patients with persistent FTMH refused to receive further surgical management. The final BCVA in the ‘LHM without retinoschisis’ group was stable compared to baseline (*P* = 0.668 by paired t-test), whereas the final BCVA of the ‘LMH with retinoschisis’ group showed marginal deterioration (0.58 ± 0.54 vs. 0.48 ± 0.37, *P* = 0.062).

**Table 2:** The summary of outcomes.

Outcome	All (112)	LMH without retinoschisis (44)	LMH with retinoschisis (68)
**Neither FD nor FTMH**	65 (58.0)	30 (68.2)	35 (51.5)
**FTMH**	19 (17.0)	9 (20.5)	10 (14.7)
**Foveal detachment**	15 (13.4)	3 (6.8)	12 (17.7)
**FTMH and MHRD**	9 (8.0)	2 (4.5)	7 (10.3)
**FD and FTMH**	2 (1.8)	0 (0.0)	2 (2.9)
**FD, FTMH, and MHRD**	2 (1.8)	0 (0.0)	2 (2.9)

FD, foveal detachment; FTMH, full-thickness macular hole; MHRD, full-thickness macular hole with retinal detachment

### Factors associated with foveal detachment or full-thickness macular hole

Univariable Cox proportional hazards regression analysis on all cases showed that the following conditions were positively associated with structural worsening: LMH with retinoschisis (*P* = 0.017), the presence of inner retinoschisis (*P* = 0.006) and outer retinoschisis (*P* = 0.029), CRT (*P* = 0.003), extensive retinoschisis (*P* < 0.001), V-shaped LMH (*P* < 0.001), and increased vertical diameter of LMH (*P* = 0.005). Elevated tissue inside the LMH (*P* < 0.001) was protective against structural worsening (Table 3). Eyes in the ‘LMH without retinoschisis’ group had no clinical features associated with structural worsening (Table 3). In eyes with LMH and retinoschisis, the presence of elevated tissue inside the LMH (*P* = 0.003) had a protective effect against structural worsening. Shorter axial length (*P* = 0.025), V-shaped LMH (*P* = 0.023), and extensive retinoschisis (*P* = 0.005) were associated with structural worsening (Table 3).

**Table 3:** Univariable regression analysis of factors associated with foveal detachment or full-thickness macular hole.

	All (112)			LMH without retinoschisis (44)			LMH with retinoschisis (68)		
Features	Hazard ratio	95% CI	*P* value	Hazard ratio	95% CI	*P* value	Hazard ratio	95% CI	*P* value
**Sex**	1.01	0.52~1.95	0.983	1.82	0.60~5.46	0.288	0.89	0.36~2.15	0.789
**Age ^a^**	0.99	0.71~1.39	0.956	0.63	0.32~1.24	0.184	1.18	0.81~1.72	0.387
**LogMAR visual acuity**	1.32	0.62~2.82	0.468	0.98	0.20~4.85	0.980	1.23	0.50~3.00	0.650
**Axial length**	0.90	0.77~1.05	0.174	0.93	0.69~1.24	0.619	0.74	0.57~0.96	**0.025**
**Type of LMH ^b^**	2.17	1.16~4.23	**0.017**	Not applicable			Not applicable		
**V-shaped LMH ^c^**	4.01	1.74~9.71	**<0.001**	2.04	0.98~4.25	0.057	3.95	1.20~12.98	**0.023**
**Inner retinoschisis**	2.28	1.27~4.09	**0.006**	Not applicable			1.76	0.87~3.59	0.118
**Outer retinoschisis**	2.03	1.08~3.84	**0.029**	Not applicable			0.40	0.12~1.32	0.131
**Extensive retinoschisis**	3.38	1.84~6.20	**<0.001**	Not applicable			2.89	1.37~6.06	**0.005**
**Epiretinal membrane**	0.82	0.46~1.48	0.508	0.62	0.21~1.85	0.393	0.82	0.40~1.68	0.592
**LHEP**	0.82	0.45~1.49	0.515	1.68	0.56~5.04	0.356	0.60	0.29~1.27	0.182
**Ellipsoid zone disruption**	1.58	0.89~2.82	0.121	1.26	0.42~3.79	0.681	1.81	0.91~3.60	0.090
**Staphyloma**	0.71	0.37~1.37	0.309	0.39	0.13~1.18	0.096	0.40	0.14~1.16	0.091
**Elevated tissue inside the LMH**	0.16	0.06~0.44	**<0.001**	0.14	0.02~1.10	0.061	0.16	0.05~0.53	**0.003**
**Vitreomacular traction**	0.62	0.29~1.35	0.226	0.65	0.17~2.43	0.523	0.69	0.26~1.82	0.449
**Myopic atrophic maculopathy**	1.15	0.64~2.08	0.643	0.88	0.29~2.64	0.817	1.10	0.53~2.27	0.801
**Central retinal thickness (μm) ^d^**	1.47	1.14~1.89	**0.003**	1.48	0.73~3.02	0.279	1.30	0.91~1.86	0.145
**Residual foveal thickness (μm) ^d^**	1.39	0.71~2.72	0.344	1.40	0.26~7.62	0.698	1.32	0.65~2.69	0.440
**Subfoveal choroidal thickness (μm) ^d^**	1.37	0.57~3.29	0.487	2.94	0.75~11.60	0.123	2.07	0.50~8.52	0.315
**Horizontal diameter (μm) ^d^**	1.05	0.87~1.27	0.615	1.09	0.77~1.55	0.630	1.08	0.87~1.34	0.486
**Vertical diameter (μm) ^d^**	1.48	1.13~1.94	**0.005**	1.22	0.59~2.51	0.587	1.35	0.92~1.99	0.125

^a^ Per 10 year

^b^ reference group: LMH without retinoschisis

^c^ reference group: A-shaped LMH

^d^ Per 100 μm

LHEP, Lamellar hole-associated epiretinal proliferation; LMH, lamellar macular hole

Multivariable Cox proportional hazards regression analysis on all cases showed that elevated tissue inside the LMH (*P* = 0.003, hazard ratio = 0.20) was protective against structural worsening and that V-shaped LMH (*P* = 0.006, hazard ratio = 3.77) predicted it (Table 4). Eyes in the ‘LMH without retinoschisis’ group had no clinical features associated with structural worsening (Table 4). In eyes with LMH and retinoschisis, ellipsoid zone disruption (*P* = 0.035, hazard ratio = 2.28), and V-shaped LMH (*P* = 0.014, hazard ratio = 4.56) were associated with structural worsening, while elevated tissue inside the LMH (*P* = 0.028, hazard ratio = 0.25) was protective against structural worsening (Table 4).

**Table 4:** Multivariable regression analysis of factors associated with foveal detachment or full-thickness macular hole.

All (112)				LMH without retinoschisis (44)				LMH with retinoschisis (68)			
Features	HR	95% CI	*P*	Features	HR	95% CI	*P*	Features	HR	95% CI	*P*
**V-shaped LMH ^a^**	3.77	1.47~9.66	**0.006**	V-shaped LMH ^a^	1.82	0.46~7.28	0.397	V-shaped LMH ^a^	4.56	1.36~15.28	**0.014**
**Elevated tissue inside LMH**	0.20	0.07~0.59	**0.003**	Elevated tissue inside LMH	0.17	0.02~1.38	0.097	Elevated tissue inside LMH	0.25	0.07~0.86	**0.028**
**Vertical diameter (μm) ^b^**	1.25	0.66~2.35	0.498	Staphyloma	0.42	0.13~1.36	0.148	Staphyloma	0.80	0.24~2.67	0.714
**Extensive retinoschisis**	1.66	0.69~3.99	0.254					Extensive retinoschisis	1.59	0.69~3.67	0.274
**Inner retinoschisis**	1.63	0.78~3.41	0.198					Ellipsoid zone disruption	2.28	1.06~4.88	**0.035**
**Outer retinoschisis**	0.30	0.03~2.59	0.274					Axial length	0.89	0.66~1.19	0.423
**Type of LMH ^c^**	1.98	0.22~17.82	0.541								
**Central retinal thickness (μm) ^b^**	1.22	0.71~2.10	0.468								

^a^ reference group: A-shaped LMH

^b^ per 100 μm

^c^ reference group: LMH without retinoschisis

HR, hazard ratio; LMH, lamellar macular hole

A subgroup analysis showed that, in eyes with extensive retinoschisis (46 eyes), V-shaped LMHs (29 out of 33 eyes) tended to have structural worsening more than A-shaped LMHs (0 out of 13 eyes) (*P* < 0.001 by Chi-squared test); in contrast, in eyes with parafoveal retinoschisis (22 eyes), V-shaped LMHs (0 out of 10 eyes) were not prone to structural worsening compared to A-shaped LMHs (3 out of 12 eyes) (*P* = 0.221, Fisher’s exact test). Among V-shaped LMHs with retinoschisis (43 eyes), those with structural worsening showed a more acute LMH angle (37.7 ± 11.9 vs. 48.4 ± 19.7, *P* = 0.050) and a lower ratio of LMH opening to LMH height (0.69 ± 0.27 vs. 1.12 ± 0.50, *P* < 0.001).

## Discussion

In non-myopic eyes, LMH in most patients has a stable clinical course, and only rarely progresses to FTMH (1%–4%) [5, 17]. However, in high myopic eyes, complex tractional forces may induce various macular structural changes, which have been collectively called MTM. LMH with retinoschisis is a specific type of MTM with inner retinal defect, which further weakens the macular structure. Only a few studies have investigated LMH in high myopia, and these yielded conflicting data [5, 9, 18]. In the present study, we found that LMHs in high myopia were unstable and took different evolution routes from those in non-myopic eyes. We identified certain factors associated with the development of FD or FTMH, which was widely regarded as the major lesions associated with structural worsening.

In the ‘LMH without retinoschisis’ group, we documented the following LMH evolution processes in highly myopic eyes: (1) stable, (2) development of macular retinoschisis, (3) development of FD, (4) direct progression to FTMH. Among these pathways, the risk of FD was the lowest, because FD occurred mostly after retinoschisis formation, and most eyes in the ‘LMH without retinoschisis’ group did not develop retinoschisis during follow-up. Even in eyes that did eventually develop macular retinoschisis during follow-up, we found that the retinoschisis was limited in extent, and that none of the eyes developed subsequent FD. Most eyes (11 out of 14 eyes, 78.6%) with structural worsening developed FTMH. The risk of developing FTMH (25%) was much higher than patients with idiopathic LMH (1%–4%) [5, 17]. Similar as our previous report [4], the progression of LMH to FTMH may have achieved through different mechanisms in this group; it may come from (1) tangential premacular traction due to epiretinal membrane (Fig 1L), (2) abnormal vitreofoveal anteroposterior traction (Fig 1N), and (3) macular atrophy with underlying scar.

Patients in the ‘LMH and retinoschisis’ group were more likely to be women and to have a longer axial length, thicker CRT, thinner subfoveal choroidal thickness, and deeper vertical extension of LMH. These features were similar to those found in MTM generally [1, 19, 20]. In this group, we found that several evolution sequences occurred: (1) stable, (2) progression to FD, or (3) development of FTMH. Importantly, the present study revealed that LMH with retinoschisis had a more unstable clinical course compared to MTM (progression rate: 48.5% vs. 17%–28%) [13, 21]. As described in the literature [4, 6], the evolution of LMH to FD or FTMH in the present study can be further classified into two patterns: (1) formation of focal elevation of the outer retinal layer, leading to FD, with subsequent FTMH formation after rupture of the outer retina; (2) widening and/or deepening of the LMH, leading to FTMH directly. Patients who developed FD were prone to progression into FTMH, so most of them (12 out of 16 eyes, 75%) underwent surgery at this stage. It follows that this early surgical intervention may explain the relatively preserved visual function in the ‘LMH and retinoschisis’ group.

We aimed to find predictive factors for structural worsening to guide follow-up planning and the timing of surgery. Multivariable regression analysis on all cases showed that V-shaped LMH was associated with structural worsening, while elevated tissue inside the LMH was a strong protective factor. The results seemed to suggest that the floor of V-shaped LMHs bore more direct traction than that of A-shaped LMHs. On the other hand, elevated tissue on the floor of the LMH may provide cushion to tractional force. The origin of this elevated tissue is unclear. In some cases, it may be the residual outer retina, or it may contain the same cellular components as lamellar hole-associated epiretinal proliferation, which represent either Müller cell proliferation or glial proliferation driven by Müller cells [12]. The presence of this elevated tissue within the LMH rendered the floor thicker and may prevent a full-thickness floor defect. One recent study involving maculopathy in high myopia reported that thinner macular choroidal thickness may be associated with myopic atrophic and neovascular maculopathy, but not with MTM [11]. The present data further showed that the baseline subfoveal choroidal thickness failed to predict the development of FD or FTMH.

In the ‘LMH with retinoschisis’ group, V-shaped LMH and elevated tissue inside the LMH were also important predictive factors. Subgroup analysis showed that the effect of V-shaped LMH on structural worsening was more significant in eyes with extensive retinoschisis. We postulated that the presence of extensive retinoschisis signified severe traction and weakened structure; these factors amplified the effect of the shape of LMH on the macular structure. Similarly, V-shaped LMH that demonstrated a narrower opening and an more acute angle were more likely to develop FD or FTMH, possibly because the tractional force was concentrated more directly on the LMH floor. Conversely, LMHs that initially presented with an A-shaped configuration, or those that transitioned from V-shaped to A-shaped in the retinoschisis group, were much less likely to progress to FD or FTMH. We hypothesized that in A-shaped LMH, either the disrupted schistic column partially released the traction or the tractional forces were distributed away from the retinal floor. Multivariable regression analysis also revealed that disrupted ellipsoid zone was associated with structural worsening. Eyes with disrupted ellipsoid zone initially had more weakened floor and was prone to structural worsening.

In contrast, among eyes in the ‘LMH without retinoschisis’ group, no clinical features or OCT parameters could predict structural worsening. Elevated tissue inside the LMH showed marginal protection from structural worsening. These patients might have less risk of progression. However, all patients should be monitored frequently to detect possible complications and to ensure timely surgical intervention.

Govetto et al. had classified idiopathic LMH into tractional and degenerative types based on their shapes and clinical characteristics [22]. In the current study population of high myopia, although some cases had “top hat” or “moustache” configuration, we found that other clinical characteristics (ellipsoid zone disruption, the presence of lamellar hole-associated epiretinal proliferation or epiretinal membrane) were different from what Govetto et al. have described. We believed that LMH in highly myopic eyes were mainly the results of complex traction. And the shape of LMH may depend on the direction and chronicity of the tractional force [23].

The present study had certain limitations because it was retrospective in nature. For instance, the actual time of FD or FTMH development could not be determined precisely. The strengths of the study were that we performed OCT regularly at intervals of 3–6 months, which increased the detection rate and that the follow-up period was longer than in previous studies.

In the present study, LMHs in highly myopic eyes had an unstable clinical course and were at risk of developing to FD or FTMH. Specifically, nearly 40% of these eyes developed FD or FTMH and required vitrectomy during a median follow-up of 67 months. Eyes with extensive retinoschisis and V-shaped LMH had more concentrated and larger traction directed towards the floor, and this was associated with structural worsening. Elevated tissue within the LMH, which may have a reparative or cushioning function, was a strong protective factor. While eyes that had LMH with retinoschisis were at higher risk of FD or FTMH than those with MTM in general, LMH without retinoschisis may progress into FTMH without obvious warning signs. Therefore, frequent follow-up in highly myopic eyes with LMH is warranted.
